# Individual-level precision diagnosis for coronavirus disease 2019 related severe outcome: an early study in New York

**DOI:** 10.1038/s41598-023-35966-z

**Published:** 2023-07-13

**Authors:** Chaorui C. Huang, Hong Xu

**Affiliations:** 1 Division of Disease Control, New York City Department of Health and Mental Hygiene, 42-09 28th St, Long Island City, NY 11101 USA; 2 Department of Neurobiology, Care Sciences and Society, Karolinska Institute, Stockholm, Sweden; 3 Department of Medical Epidemiology and Biostatistics, Karolinska Institute, Stockholm, Sweden

**Keywords:** Infectious diseases, Biomarkers, Diagnostic markers, Predictive markers, Prognostic markers

## Abstract

Because of inadequate information provided by the on-going population level risk analyses for Coronavirus disease 2019 (COVID-19), this study aimed to evaluate the risk factors and develop an individual-level precision diagnostic method for COVID-19 related severe outcome in New York State (NYS) to facilitate early intervention and predict resource needs for patients with COVID-19. We analyzed COVID-19 related hospital encounter and hospitalization in NYS using Statewide Planning and Research Cooperative System hospital discharge dataset. Logistic regression was performed to evaluate the risk factors for COVID-19 related mortality. We proposed an individual-level precision diagnostic method by taking into consideration of the different weights and interactions of multiple risk factors. Age was the greatest risk factor for COVID-19 related fatal outcome. By adding other demographic variables, dyspnea or hypoxemia and multiple chronic co-morbid conditions, the model predictive accuracy was improved to 0.85 (95% CI 0.84–0.85). We selected cut-off points for predictors and provided a general recommendation to categorize the levels of risk for COVID-19 related fatal outcome, which can facilitate the individual-level diagnosis and treatment, as well as medical resource prediction. We further provided a use case of our method to evaluate the feasibility of public health policy for monoclonal antibody therapy.

## Introduction

Given the heterogeneous clinical presentation and outcomes of people acutely ill with severe acute respiratory syndrome coronavirus 2 (SARS-CoV-2) or Coronavirus 2019 (COVID-19), and the scope of the outbreak, there is an urgent need to develop a risk stratification tool for COVID-19^1–5^. This system can be used to identify high risk patients for early treatment and medical intervention, be used to flag and track patients who are at high risk for deterioration upon hospitalization, and can be used to accurately allocate resources and staff for outbreak response.

The immediate application of this risk stratification method is to support monoclonal antibody therapy and anti-viral therapies, which were mainly provided for the high-risk COVID-19 patients. But it was unclear exactly who should be prioritized for such early intervention. Without this knowledge, it is also difficult to calculate the daily medical supply.

The on-going research has discovered multiple risk factors for COVID-19 related severe outcome, which mainly included age and a long list of co-morbid conditions^6–8^. However, most of these epidemiology research studies primarily focused on the population-level results, such as population probability and risk/odds ratio, which are hard to interpret in clinical settings. It is important to clarify that population risk isn’t equivalent to individual risk, and the population risk cannot be directly applied in clinic to treat individual patient. In other words, population risk doesn’t necessarily indicate that every single patient is also at risk. One of the main reasons for that is because most of the population studies didn’t well control the confounding factors. For example, in an analysis of nearly 300,000 confirmed COVID-19 cases reported in the United States, the study reported that the mortality rate was 12 times as high among patients with co-morbidities compared with those with none^9^. However, age has also been identified as a risk factor for COVID-19 related fatal outcome by population studies, and it was well documented that there was an association between increasing age and chronic disease occurrence^6,10^. Therefore, it was hard to tell from these population results regarding to what was the true cause driving the increased mortality rate—age or co-morbid conditions or both?

Another concern for population risk factor is that different risk factors could have different weights in determining the patients’ outcomes. In other words, age may be a more important risk factor than co-morbid conditions, or vice versa. However, treatment regimen guided by the population risk factors does not reflect the weight difference, instead, it considered every risk factor equally important and treat the patients with different risk factors the same way.

The population-based risk studies also do not provide information regarding to how the multiple risk factors with different combinations in a single patient interact with each other and how the interactions can affect the patient’s outcome. For example, it is not clear how a 75-year-old patient with 3 co-morbid conditions (i.e. diabetes, hypertension and cardiovascular disease) differs from a 35- year-old patient with diabetes only. The population risk factor guided treatment regimen will treat both patients the same way. The question is that—are these two patients really the same? Do they really have the same chance of developing the severe outcome? Intuitively, it does not seem to be. But the population risk factor guided treatment regimen cannot make such distinction.

Unfortunately, population risk factor guided treatment regimen is nowadays widely implemented in clinical practice to treat individual patient. Every patient who is under population risk will be mistakenly considered as having individual risk, and therapeutical intervention will be given to these patients equally. Scientific community and medical specialists are not aware this is a problem. What we need urgently in clinic is a system to identify the individual risk factors to support the development of diagnostic process and treatment regimen.

In this study, we aimed to evaluate the individual patient’s risk factors for COVID-19 related severe outcome, specifically in-hospital death, in New York State (NYS) and propose a strategy, which can be directly applied to clinics to rapidly screen the individual at-risk patients for early intervention. It will also aid the daily clinical operation, such as medical supply calculation, as well as resource and staff allocation.

## Methods

### Data source and study population

We analyzed Statewide Planning and Research Cooperative System (SPARCS) hospital discharge data for NYS residents (based on address of home residence) who were either hospitalized or visited ambulatory surgery or emergency department or outpatient, because of COVID-19, from April 1st to November 17th, 2020. We also conducted post-hoc analysis in two separated sub-samples in New York City (NYC), which included the five boroughs of Manhattan, Queens, Bronx, Brooklyn, and Staten Island, and in other NYS regions.

SPARCS is a comprehensive all payer data reporting system that collects discharge data from all hospitals in NYS^11^. Each discharge record within SPARCS includes a principal diagnosis and multiple secondary diagnoses, coded using the *International Classification of Diseases, 10th revision, Clinical Modification* (*ICD-10-CM)*^12^.

### Variables, covariates and outcome

We identified COVID-19 related hospitalizations and hospital visits by examining the principal diagnosis. Of these records, we further identified mortality, which served as the main study outcome. Covariates include age, sex, race/ethnicity, location, clinical presentation/examination and chronic co-morbid conditions. The clinical presentation/examination and chronic co-morbid conditions included dyspnea or hypoxemia, overweight or obesity, essential (primary) hypertension, diabetes mellitus, hyperlipidemia, chronic cardiovascular disease, chronic kidney disease, chronic pulmonary disease, malignant neoplasms, dementia, human immunodeficiency virus (HIV), cerebral palsy, sickle-cell disorders, asthma, nicotine dependence, and pregnancy. The clinical presentation/examination and chronic co-morbid conditions were selected by manual review of the secondary diagnosis of COVID-19 related hospital encounter, as well as the risk profile provided by Centers for Disease Control and Prevention (CDC)^6^.

### Data analysis

The unit of analysis was a hospitalization or hospital visit at ambulatory surgery or emergency department or outpatient. This study was initially conducted in the NYC population, then the results were further validated in the population in other NYS regions. For the final report, we combined the NYC and other NYS regions sample.

We firstly conducted the descriptive statistics and calculated the count of in-hospital death, and length of hospital stay in NYS. We then performed multivariate logistic regression to evaluate the risk factors for the COVID-19 related severe outcome (mortality). A total of 15 subjects were removed from the modeling process due to small sample size in the category of “sex = other”. We built up two logistic regression models, which were “age model” and “all effect model”. The outcome was COVID-19 related mortality status. The predictors in all effect model were demographic variables (age, sex, race/ethnicity, location), clinical presentation/examination (dyspnea or hypoxemia), and chronic co-morbid conditions. Of note, race/ethnicity itself is not a predictor for severe COVID-19 outcomes, but rather a proxy for unmeasured social context/factors, including structural vulnerability and racism. We calculated ROC curve for each model, as well as the Brier score. We then calculated the predicted odds and probability for the outcome among the individual subjects, and generated sensitivity and specificity table. Based on the sensitivity and specificity table, we selected the cut-off points of odds and probability, and provided a general recommendation to stage the risk of fatal outcome among the COVID-19 patients^13^. We further developed an individualized predictive model for individual patient’s risk prediction.

We further presented a use case by applying the model results to evaluate the benefit and cost of monoclonal antibody therapy, Sotrovimab intravenous, 500 mg/8 mL, for early treatment of COVID-19 related mortality at-risk patients. The cost of Sotrovimab was set as $315.00 per patient, and total cost including medication, cost for hospital and infusion center administration, and medical staffs was set as $2,000.00 per patient.

We then compared the results from our precision diagnostic approach with the results from the population risk-based treatment regimen. The treatment regimen guided by the population risk was defined as such, that every COVID-19 patient ≥ 65 years old, and/or with at least one chronic co-morbid condition will be given treatment of Sotrovimab to prevent fatal outcome. The co-morbid conditions included overweight or obesity, essential hypertension, diabetes mellitus, chronic cardiovascular disease, chronic kidney disease, chronic pulmonary disease, malignant neoplasms, dementia, HIV, cerebral palsy.

All statistical analyses were performed using SAS, version 9.4, SAS Institute, and R (https://www.r-project.org/).

### Study approval

This activity was determined by NYC Department of Health and Mental Hygiene (DOHMH) to involve the use of existing data and consent form was not required for individual subjects. The data included every inpatient hospital discharge, ambulatory surgery visit, emergency department admission and outpatient visits from health care facilities certified under Article 28 of the New York State Public Health Law. This study was exempt from DOHMH Institutional Review Board review.

## Results

### Descriptive statistics

From April 1st to November 17th in 2020, there were 102,440 COVID-19 hospitalizations or visits at ambulatory surgery or emergency department or outpatient in total in NYS, among which 61,296 (59.8%) were from NYC, and 41,144 (40.2%) were from other regions in NYS. Majority of deaths (10,091) occurred in hospitals, and 2 cases occurred in medical facilities for hospice care. No death was recorded at home or at other places. There was no missing data for discharge disposition. We therefore referred the outcome of this study as in-hospital death. The overall COVID-19 related percentage of in-hospital death in NYS was 9.9%. The overall COVID-19 related percentage of in-hospital death in NYS was 0.3% among children less than 18 years old; 3.8% among adults from 18 to 65 years old, and 20.9% among elderly older than 65 years old. A total of 97.1% of the in-hospital death occurred among the hospitalized patients, and 2.9% at emergency department. The median length of hospital stay was 6 days (Interquartile Range: 3–11 days) days.

### Prediction of COVID-19 related in-hospital death

The results of maximum likelihood estimate of logistic regression and odds ratio for COVID-19 related severe outcome in NYS were shown in Table 1.

**Table 1:** Logistic Regression Estimates of the Risk Factors of COVID-19 related in-Hospital Death in NYS.

Parameter	Estimate (β)	Standard Error	Wald Chi-Square	Pr > ChiSq	Odds Ratio (CI)
Intercept	− 7.2813	0.0767	9001.5299	< .0001	–
Age (Year)	0.0528	0.000966	2981.4721	< .0001	1.05 (1.05–1.06)
Sex (Male vs. Female)	0.4264	0.0242	310.0159	< .0001	1.53 (1.46–1.61)
Non-Hispanic Black vs. Non-Hispanic White	0.2453	0.0335	53.7615	< .0001	1.28 (1.20–1.37)
Hispanic vs. Non-Hispanic White	0.2766	0.0343	64.8833	< .0001	1.32 (1.23–1.41)
Asian/Pacific Islander vs. Non-Hispanic White	0.3596	0.0532	45.6535	< .0001	1.43 (1.29–1.59)
Other Races vs. Non-Hispanic White	0.1664	0.0392	17.9916	< .0001	1.18 (1.09–1.28)
Location (NYC vs NYS)	0.4169	0.0264	249.1542	< .0001	1.52 (1.44–1.60)
Dyspnea or Hypoxemia	1.0900	0.0242	2036.6791	< .0001	2.97 (2.84–3.12)
Overweight or Obesity	0.4430	0.0396	125.2224	< .0001	1.56 (1.44–1.68)
Essential (Primary) Hypertension	0.1593	0.0282	31.8640	< .0001	1.17 (1.11–1.24)
Diabetes Mellitus	0.3798	0.0254	222.9089	< .0001	1.46 (1.39–1.54)
Chronic Cardiovascular Disease	0.1713	0.0280	37.3251	< .0001	1.19 (1.12–1.25)
Chronic Kidney Disease	0.7156	0.0335	456.2693	< .0001	2.05 (1.92–2.18)
Chronic Pulmonary Disease	0.3223	0.0378	72.6078	< .0001	1.38 (1.28–1.49)
Malignant Neoplasms	0.2222	0.0369	36.2386	< .0001	1.25 (1.16–1.34)
Dementia	0.3726	0.0339	120.5005	< .0001	1.45 (1.36–1.55)
HIV	0.2301	0.0992	5.3768	0.0204	1.26 (1.04–1.53)
Cerebral Palsy	0.8086	0.1905	18.0211	< .0001	2.25 (1.55–3.26)
	Area Under the ROC Curve (CI)				
Age Model	0.78 (0.78–0.79)				
All Effect	0.85 (0.85–0.85)				

*CI* Confidence Interval, *HIV* Human Immunodeficiency Virus.

**Figure 1:**
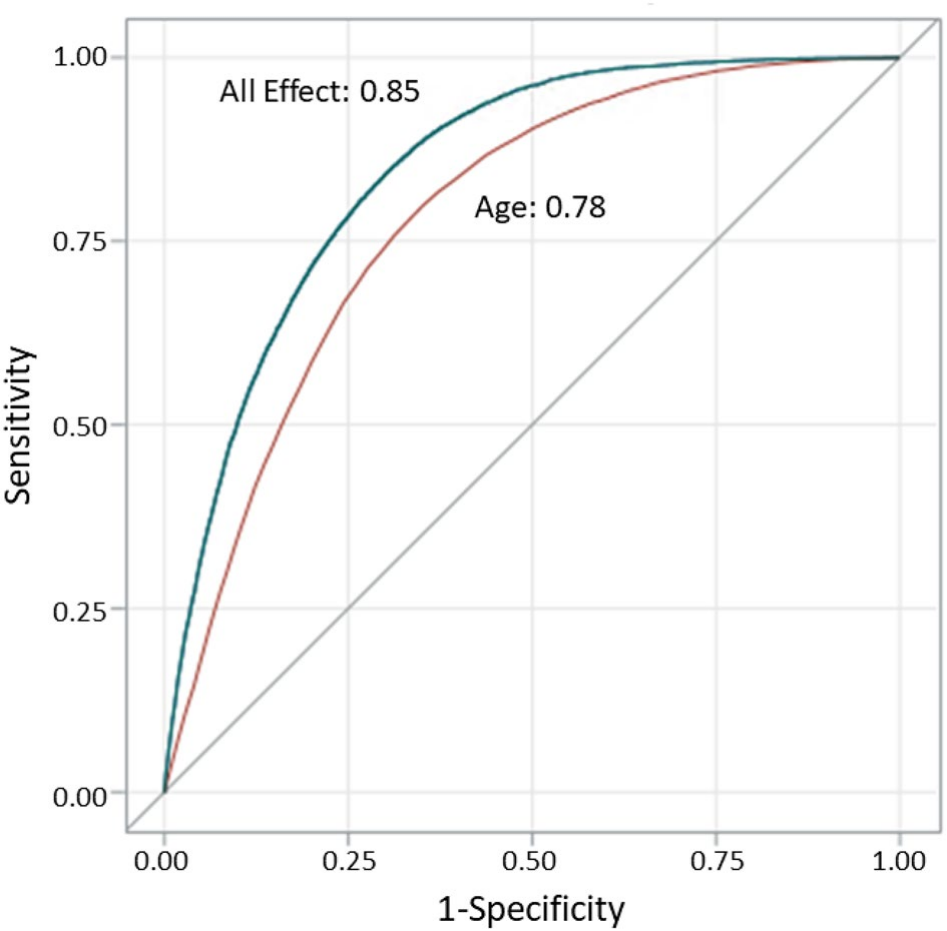
Receiver Operating Characteristic (ROC) Curve as an Estimate of Diagnostic Accuracy for COVID-19 related Fatal Outcome in NYS. Figure legend: The area under the ROC curve for age model was 0.78. The area under the ROC curve for combined effect of demographic variables (age, sex, race/ethnicity), clinical presentation/examination, and all chronic co-morbid conditions was 0.85.

The first model with only age as a predictor showed that age was a significant risk factor for COVID-19 related in-hospital death. It achieved a diagnostic accuracy of 0.78, represented by the area under the ROC curve (Table 1, Fig. 1).

In the second “all-effect" model, we added the covariates step-by-step. By including the chronic co-morbid conditions together with age, the diagnostic accuracy improved from 0.78 to 0.82. In the final model, demographic variables (age, sex, race/ethnicity, location), clinical presentation/examination (dyspnea or hypoxemia), and chronic co-morbid conditions (overweight or obesity, essential hypertension, diabetes mellitus, chronic cardiovascular disease, chronic kidney disease, chronic pulmonary disease, malignant neoplasms, dementia, HIV, cerebral palsy) were significant predictors for COVID-19 related in-hospital death. The diagnostic accuracy of this final model for predicting the COVID-19 related fatal outcome was 0.85, represented by the area under the ROC curve (Table 1, Fig. 1). The Brier score was 0.0741, which indicted a good predictive accuracy.

With further manual calculation based on the coefficient in Table 1 for “all-effect" model, the results showed that the odds of a COVID-19-related fatal outcome for 65-year-old patients was 11.9 times the odds of 18-year-old patients, and 23.6 times the odds of 5-year-old patients, after accounting for sex, race/ethnicity, location, dyspnea or hypoxemia and chronic co-morbid conditions. Patients of Asian ancestry had the highest odds for COVID-19 related fatal outcome among all races, after accounting for age, sex, location, dyspnea or hypoxemia, and chronic co-morbid conditions. The odds of a COVID-19-related fatal outcome for patients living in NYC was 1.5 times the odds of patients living in other NYS region, after accounting for age, sex, race/ethnicity, dyspnea or hypoxemia and chronic co-morbid conditions. The odds ratio of chronic co-morbid conditions for COVID-19 related fatal outcome typically ranged between 1.0 and 3.0, after correcting for demographic variables and dyspnea or hypoxemia (Table 1).

### Risk staging

The ROC curve, which evaluated how well a continuous predictor can classify a binary outcome, was plotted based on the sensitivity and specificity table. The cut-off points of the predictors (predicted odds and/or probability), which can provide the most optimal sensitivity and specificity for diagnostic classification, were evaluated. The ideal cut-off point is supposed to be the predictor value corresponding to the point on the ROC curve, which is closest to the upper left corner. In this study, with the moderate diagnostic accuracy of 0.85, we proposed two methods for cut-off point selection.

For method I, we chose the nearest point to the upper left corner of the ROC curve graph and classified the patients to high-risk group vs. low-risk group for the COVID-19 related mortality^13^. For method II, we proposed a range of cut-off points and classified the risk of the COVID-19 related in-hospital death into five stages. We arbitrarily selected four cut-off points of predictive odds and/or probability, which corresponded to the sensitivity and specificity level at 95% and 80% on the ROC curve, separately. Five levels of risk for COVID-19 related severe outcome were ranked, which were high risk for mortality, at-risk (high end) for mortality, at-risk for mortality, at-risk (low end) for mortality, and low risk for mortality (Table 2). We also provided additional cut-off points of odds and/or probability and corresponding sensitivities and specificities in Table 2. Clinicians can choose to use different cut-off points based on their own clinic needs for i.e. diagnostic or supply calculation purpose.

**Table 2:** General recommendation for staging the COVID-19 related severe outcome from all-effect model.

Staging	Predictive Odds	Probability	Interpretation
Staging recommendation			
Method I			
Stage II	≥ 0.108	≥ 0.098	High risk for mortality
Stage I	< 0.108	< 0.098	Low risk for mortality
Method II			
Stage V	≥ 0.479	≥ 0.324	High risk for mortality
Stage IV	[0.146–0.479)	[0.128–0.324)	at-risk (high end) for mortality
Stage III	[0.108–0.146)	[0.098–0.128)	at-risk for mortality
Stage II	[0.042–0.108)	[0.040–0.098)	at-risk (low end) for mortality
Stage I	< 0.042	< 0.040	Low risk for mortality

Predictive Odds	Probability	Sensitivity (%)	Specificity (%)
Selected Cut-off Points for Staging			
1.079	0.519	9.9%	99.0%
0.479*	0.324*	32.8%*	95.0%*
0.289	0.224	50.9%	90.0%
0.199	0.166	63.2%	85.0%
0.146*	0.128*	72.2%*	80.0%*
0.108*	0.098*	80.0%*	74.4%*
0.085	0.078	85.0%	69.5%
0.063	0.059	90.0%	63.0%
0.042*	0.040*	95.0%*	53.6%*
0.017	0.017	99.0%	32.3%

*Generally recommended cut-off points.

### Development of an individualized predictive model

To present with the clinicians regarding to how to use this developed algorithm for their day-to-day clinical work to predict an individual patient’s risk for COVID-19 related severe outcome, we provided a practical patient’s example in Table 3. This patient was assumed to be 55-year-old, male, Asian, lives in NYS, but outside NYC, and has essential hypertension, diabetes mellitus and chronic cardiovascular disease. Physicians can calculate this patient’s odds and probability using the formulas below. The β value for each risk factor was presented in Table 1. After the calculation, physicians can use the references presented in Table 2 to define the patient’s risk for COVID-19 related severe outcome and decide if this patient is a right candidate for early intervention or not. We also developed an interactive app to facilitate this individualized diagnosis (Supplementary Table 1).

$$\begin{aligned} & {\text{Odds}} = {\text{Exp}}\left( {\beta_{0} + \beta_{{{1}}}{*} \;{\text{risk}}\;{\text{factor}}_{{1}} + \beta_{{{2}}}{*} \;{\text{risk}}\;{\text{factor}}_{{2}} \cdots + \beta_{{{\text{n}}}}{*} \;{\text{risk}}\;{\text{factor}}_{{\text{n}}} } \right) \\ & {\text{Probability}} = {\text{Odds}}/\left( {{1} + {\text{Odds}}} \right) \\ \end{aligned}$$

**Table 3:** Development of individualized risk score practicing sheet with a patient example.

	Please enter patient information here	Instruction
Patient's Information		
Age	55	Years
Sex	1	Enter 1, if Male; Enter 0, if Female
Race (Non-Hispanic White)*	0	Enter 1, if Yes; Enter 0, if No
Race (Non-Hispanic Black)	0	Enter 1, if Yes; Enter 0, if No
Race (Asian or Pacific Islander)	1	Enter 1, if Yes; Enter 0, if No
Race (Others)	0	Enter 1, if Yes; Enter 0, if No
Location	0	Enter 1, if lives in NYC; Enter 0, if lives in NYS
Ethnicity (Hispanic)	0	Enter 1, if Yes; Enter 0, if No
Dyspnea or Hypoxemia	0	Enter 1, if Yes; Enter 0, if No
Overweight or Obesity	0	Enter 1, if Yes; Enter 0, if No
Essential (Primary) Hypertension	1	Enter 1, if Yes; Enter 0, if No
Diabetes Mellitus	1	Enter 1, if Yes; Enter 0, if No
Chronic Cardiovascular Disease	1	Enter 1, if Yes; Enter 0, if No
Chronic Kidney Disease	0	Enter 1, if Yes; Enter 0, if No
Chronic Pulmonary Disease	0	Enter 1, if patient has COPD or chronic pulmonary condition; Otherwise, enter 0
Malignant Neoplasms	0	Enter 1, if patient has cancer or history of cancer; Otherwise, enter 0
Dementia	0	Enter 1, if Yes; Enter 0, if No
HIV	0	Enter 1, if Yes; Enter 0, if No
Cerebral Palsy	0	Enter 1, if Yes; Enter 0, if No
Physician's Information		
	Odds^†,‡^	Probability^‡^
Calculation Formula*	Exp (β_0_ + β_1_*risk factor_1_ + β_2_*risk factor_2_ … + β_n_*risk factor_n_)	Odds/(1 + Odds)
A patient example: 55-year-old, Male, Asian, lives in NYS, but outside NYC, has Essential Hypertension, Diabetes Mellitus & Chronic Cardiovascular Disease	Exp (− 7.2813 + 0.0528*55 + 0.4264*1 + 0.3596*1 + 0.1593*1 + 0.3798*1 + 0.1713*1) = 0.056	0.056/(1 + 0.056) = 0.053

*For Non-Hispanic White, it does not need to be incorporated in the equation, no β value was provided in Table 1, because this category is the reference in race/ethnicity variable.

^†^β value for individual covariate was presented in Table 1.

^‡^After odds and probability are calculated, please use the cut-off point presented in Table 2 to stage the risk level for severe outcome for this patient. This example patient was categorized at stage II: at-risk (low end) for mortality.

*COPD*: Chronic Obstructive Pulmonary Disease, *HIV*: Human Immunodeficiency Virus.

### Study validation

This study was conducted in the NYC and in the other NYS regions independently for validation purpose. The results showed that the odds ratio of chronic co-morbid conditions varied, but still typically ranged between 1.0–3.0 in both samples. The diagnostic accuracy for COVID-19 related fatal outcome was also similar in both samples, with age model reached diagnostic accuracy of 0.80 and 0.77 respectively, and overall diagnostic accuracy of 0.85 in both samples, with the combined demographic variables, clinical presentation/examination, and chronic co-morbid conditions as predictors, represented by the area under the ROC curve (Table 4).

**Table 4:** Comparison of Model Estimates for COVID-19 related in-Hospital Death between NYC and Other NYS Regions.

Parameter	NYC		Other NYS Regions	
	Estimate (β)	Odds Ratio (CI)	Estimate	Odds Ratio (CI)
Intercept	− 7.0235	–	− 6.9936	–
Age (Year)	0.0567	1.06 (1.06–1.06)	0.0465	1.05 (1.04–1.05)
Sex (Male vs. Female)	0.4254	1.53 (1.44–1.62)	0.4227	1.53 (1.40–1.66)
Non-Hispanic Black vs. Non-Hispanic White	0.2286	1.26 (1.16–1.36)	0.1948	1.22 (1.08–1.37)
Hispanic vs. Non-Hispanic White	0.1699	1.19 (1.09–1.29)	0.5342	1.71 (1.51–1.92)
Asian/Pacific Islander vs. Non-Hispanic White	0.2953	1.34 (1.19–1.51)	0.4914	1.64 (1.30–2.06)
Other Races vs. Non-Hispanic White	0.1165	1.12 (1.02–1.24)	0.2260	1.25 (1.09–1.44)
Dyspnea or Hypoxemia	1.1433	3.14 (2.96–3.32)	0.9437	2.57 (2.36–2.79)
Overweight or Obesity	0.3930	1.48 (1.34–1.63)	0.5328	1.70 (1.50–1.94)
Essential (Primary) Hypertension	0.0799	1.08 (1.01–1.16)	0.2948	1.34 (1.22–1.48)
Diabetes Mellitus	0.3955	1.49 (1.40–1.58)	0.3380	1.40 (1.28–1.53)
Chronic Cardiovascular Disease	0.0990	1.10 (1.03–1.18)	0.3019	1.35 (1.23–1.49)
Chronic Kidney Disease	0.5749	1.78 (1.64–1.93)	0.9946	2.70 (2.41–3.03)
Chronic Pulmonary Disease	0.2625	1.30 (1.18–1.43)	0.4110	1.51 (1.34–1.70)
Malignant Neoplasms	0.1787	1.20 (1.09–1.31)	0.2963	1.35 (1.19–1.52)
Dementia	0.3004	1.35 (1.24–1.47)	0.4961	1.64 (1.47–1.83)
HIV	0.2826	1.33 (1.08–1.63)	0.1446	1.16 (0.65–2.06)
Cerebral Palsy	1.0654	2.90 (1.74–4.84)	0.5840	1.79 (1.02–3.14)
	Area Under the ROC Curve (CI)		Area Under the ROC Curve (CI)	
Age Model	0.80 (0.79–0.80)		0.77 (0.77–0.78)	
All Effect	0.85 (0.84–0.85)		0.85 (0.84–0.86)	

*CI* Confidence Interval.

### A use case to evaluate the feasibility of public health policy

We used Method I cut-off point (probability = 0.098) presented in Table 2 to calculate benefit and cost of Sotrovimab intravenous treatment for COVID-19 related severe outcome at-risk patients. We then compared the results with the treatment regimen guided by the population risk.

The treatment regimen developed based on the population risk factors resulted in high sensitivity (95.5%), but very low specificity (47.1%), and overall diagnostic accuracy of 51.9%. The precision diagnostic regimen we developed in this study had improved overall diagnostic accuracy of 75.0% (Table 5).

**Table 5:** Treatment Regimen for at-risk COVID-19 Patients for Severe Outcome and Corresponding Diagnostic Accuracy, Benefits and Costs.

Results	Precision Treatment Regimen at Individual Level	Population Level Treatment Regimen
True Positive	8068	9638
True Negative	68734	43506
False Positive	23600	48828
False Negative	2023	453
Sensitivity (95% CI)	80.0% (79.2–80.7%)	95.5% (95.1–95.9%)
Specificity (95% CI)	74.4% (74.2–74.7%)	47.1% (46.8–47.4%)
Diagnostic Accuracy	75.0% (74.7–75.3%)	51.9% (51.5–52.2%)
Cost for Medicine ($)^a^	9,975,420	18,416,790
Total Cost ($)^b^	63,336,000	116,932,000

^a^Use monoclonal antibody, Sotrovimab intravenous, 500 mg/8 mL, as an example, which costs $315.00 per patient. Cost for Medicine ($) = (True Positive + False Positive)*315.

^b^A total cost of $2,000.00 per patient including the cost for hospital and infusion center administration and medical staffs. Total Cost ($) = (True Positive + False Positive)*2000.

The treatment regimen based on the population risk factors prevented 1,570 more patients from fatal outcome than the individual-based precision treatment regimen. However, it added 8.4 million dollars of cost for Sotrovimab, and overall additional 53.6 million dollars cost, including the cost for hospital and infusion center administration and medical staffs (Table 5).

## Discussion

In this study, we evaluated the clinical risk factors for COVID-19 related severe outcome and developed an algorithm for precision diagnosis targeting the patients at an individual level, instead of using the population results to guide the individual patient’s early intervention, which can be largely unspecific. The algorithm we developed in the current study took into consideration of different weights of risk factors, and the effect of the different combinations of multiple risk factors in a single patient. We also provided physicians with step-by-step practical guidance and recommendation to categorize the level of risk for COVID related severe outcome. In addition, we demonstrated the utility of this precision model for public health policy evaluation.

Physicians can calculate an individual COVID-19 patient’s odds or probability (they can be derived from each other) of developing fatal outcome, using the estimates (β) provided in Table 1 and logistic regression formula shown in the Results section and Table 3. Based on the recommended reference we provided in Table 2, they can further define each individual patient’s risk for severe outcome and decide if the patient is a suitable candidate for the early intervention, such as monoclonal antibody therapy or antiviral therapy, or not. We have also developed an interactive app to facilitate this pre-clinical diagnosis process, which was shown in Supplementary Table 1.

Among all the risk factors we discovered in this study for COVID-19 related severe outcome, age may be the most important one, which was consistent with a previous study^16^. We showed in the study that age by itself can achieve 0.77–0.80 diagnostic accuracy, represented by the area under the ROC curve. All the multiple co-morbid conditions together improved around 4% of diagnostic accuracy on top of age. In addition, the odds ratio of the co-morbid conditions typically ranged between 1.0 and 3.0, after correcting for demographic variables and dyspnea or hypoxemia. However, the odds of a COVID-19 related fatal outcome increased with much greater magnitude with increased age, such that the odds of 65-year-old patients was 11.9 times that of 18-year-old patients, and 23.6 times that of 5-year-old patients for fatal outcome, after correcting for other demographic variables, dyspnea or hypoxemia, and chronic co-morbid conditions.

However, these findings do not necessarily mean that the co-morbid conditions weren’t important and should not be considered in decision making for a treatment option, but rather indicated that age had greater weight in determining the patients’ fatal outcome. The association between age and many co-morbid conditions has been well established for decades.^10^ For the predictive purpose, the combined multiple co-morbid conditions without age may also suffice, though age, as a single variable, is likely much easier to manage in clinics.

The main strength of this study was that we demonstrated the method of cut-off point selection for individual precision diagnosis in this study. Most of the current on-going population-based epidemiology studies only calculated predicted probability at population level. But the predicted probability calculated from such as logistic regression model, was a continuous variable, which didn’t directly reflect the binary outcome, such as mortality status, and also cannot be used to classify patients at individual level^17^. In order to use it as a classifier, and have it applied to individual patient, a cut-off value needs to be chosen.

In this study, if we were able to achieve an ideal diagnostic accuracy (e.g., sensitivity and specificity above 95%), we would suggest one single cut-off point of odds and/or probability for classification purpose, which should reside on the ROC curve closest to the upper left corner. However, since the diagnostic accuracy was moderate (0.85) in the study, to better facilitate the clinical operation, we proposed two methods for cut-off point selection. For method I, we chose one single cut-off point of odds and/or probability, which was on the ROC curve closest to the upper left corner and classified the patients to two categories, which were high risk vs. low risk for the COVID-19 related fatal outcome (Table 2). In method II, we proposed a range of cut-off points based on the sensitivity and specificity table and provided a general recommendation to stage the risk to five levels (Table 2). However, physicians can choose a different cut-off point of odds and/or probability based on their clinic needs for patient management.

Several previous studies have published COVID-19 related fatal outcome predictive model^14,15^. But they used machine learning methods, and the parameters can be hard to interpret. We chose to use logistic regression model in this study over other machine learning predictive models, which was because systematic review showed no performance benefit of machine learning over logistic regression for clinical prediction^18^. The parameters of logistic regression is also much easier to interpret than the machine learning methods in general. In addition, we also conducted and validated the results in two independent samples, which were NYC population and other NYS region population.

In the use case of monoclonal antibody therapy of Sotrovimab, we demonstrated the utility of our precision approach in supporting the evaluation of the public health policy. The population risk factors so far have been widely implemented to instruct the clinical practice for diagnosis and treatment regimen for COVID-19 patients, who are at-risk to develop severe outcome. However, our study showed that it had resulted in a large amount of patient misclassification, with only 51.9% overall diagnostic accuracy, which is close to a random chance. Also, by preventing 1,570 more patients from fatal outcome, it can add additional 53.6 million dollars total cost (Table 5). Beside that, we also need to consider the maximum capacity of hospital and infusion center. It may potentially collapse the healthcare system by treating that many misclassified patients within the short treatment time window. Furthermore, the healthcare system does not only treat COVID-19 patients. There are also demands from other critical diseases and surgeries for healthcare service, which could also potentially lead to a fatal outcome.

Using the precision approach we developed in this study, it improved the diagnostic accuracy to 75%, which can help the medical specialists to more accurately identify the right patients to treat, and properly allocate the medical supplies. By adding laboratory biomarkers in the model, we hope the diagnostic accuracy can be further improved in future studies.

However, several limitations should be also taken into consideration. Firstly, these data were collected relatively early during the SARS-CoV-2 pandemic and before the predominant omicron variant emerged. However, the current methodology is still likely to be valid with the new variants, as well as the changes of vaccination status. It is a matter of updating the cut-off point threshold and implement vaccination status into the model with the newer datasets. Secondly, it may also be necessary to evaluate in larger samples, if the co-morbid conditions matter more among children and young adults than elderly in terms of their associations with the fatal outcome. Thirdly, it would be better to use case incidence as covariate in the model, instead of incidence of hospitalization as a proxy, since viral load exposure has been linked to fatality independent of other covariates. Lastly, it is also important to mention that clinical measurements can vary significantly and are often unable to offer a very high-level diagnostic accuracy in predicting future outcomes. Biomarker development combined with the clinical measures has been going on in many fields for decades to facilitate the clinical outcome predictions and evaluate the pharmaceutical intervention for new drugs, which may be more reliable indicators than the clinical presentation and medical history alone^19–23^.

To conclude, our study showed that age and chronic co-morbid conditions are risk factors for COVID-19 related fatal outcome. In addition, we developed an algorithm that took into consideration of the different weights of risk factors targeting the patients at an individual level and evaluated its utility for public health policy. Further studies are warranted to develop laboratory biomarkers and evaluate the clinical assessment in combination with laboratory tests to improve the diagnostic accuracy and longitudinal prediction, so that at-risk patients can be identified at an early stage for intervention with the improved outcome.

### Supplementary Information

Supplementary Information.

## Data Availability

The datasets analyzed during the current study are available in the SPARCS repository. https://www.health.ny.gov/statistics/sparcs/.
