# Author Correction: Individual-level precision diagnosis for coronavirus disease 2019 related severe outcome: an early study in New York

**DOI:** 10.1038/s41598-023-40792-4

**Published:** 2023-09-20

**Authors:** Chaorui C. Huang, Hong Xu

**Affiliations:** 1 Division of Disease Control, New York City Department of Health and Mental Hygiene, 42-09 28th St, Long Island City, NY 11101 USA; 2 Department of Neurobiology, Care Sciences and Society, Karolinska Institute, Stockholm, Sweden; 3 Department of Medical Epidemiology and Biostatistics, Karolinska Institute, Stockholm, Sweden

Correction to: *Scientific Reports* https://doi.org/10.1038/s41598-023-35966-z, published online 13 July 2023

The original version of this Article contained an error in Affiliation 1, which was incorrectly given as ‘Division of Disease Control, New York City Department of Health and Mental Hygiene, Bureau of Public Health Clinic, 42-09 28th St, Long Island City, New York City, NY, 11101, USA’. The correct affiliation is listed below:

Division of Disease Control, New York City Department of Health and Mental Hygiene, 42-09 28th St, Long Island City, NY 11101, USA.

The original Article has been corrected.

