# Supplementary material for: Individual-level precision diagnosis for coronavirus disease 2019 related severe outcome: an early study in New York
Source: Sci Rep. 2023 Jul 13;13:11317. doi: 10.1038/s41598-023-35966-z (PMC10344938; doi:10.1038/s41598-023-35966-z)
Supplement: Supplementary file 1 — Supplementary Information. [file 41598_2023_35966_MOESM1_ESM.docx]

**Supplementary Table 1. Interactive App for Individualized Risk Score Calculation
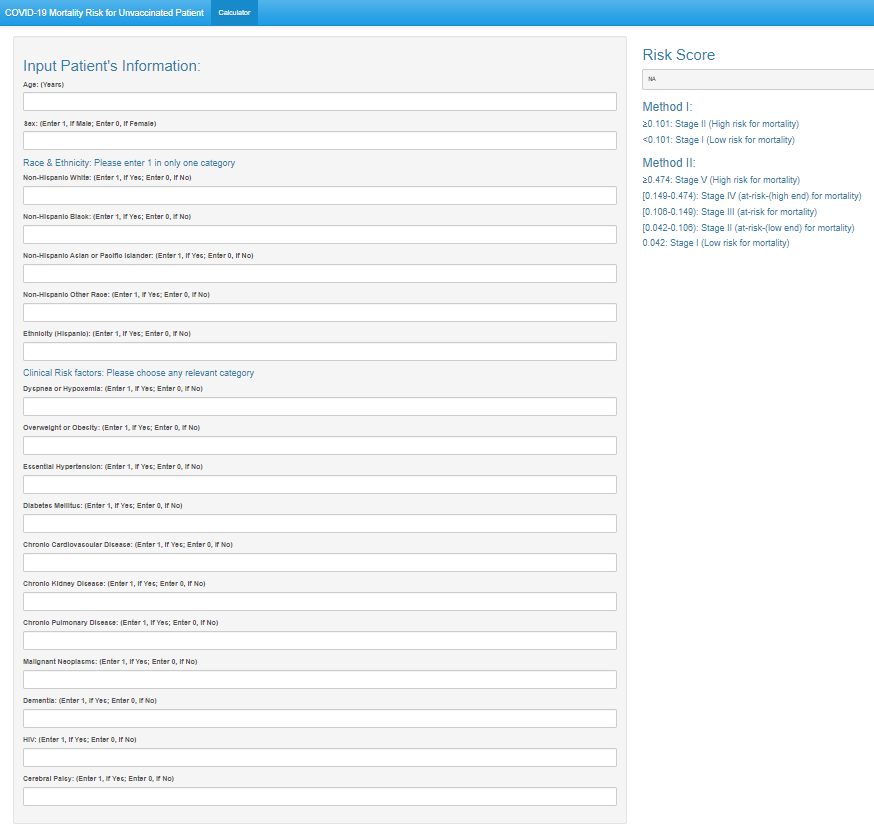
**

*R code for the interactive app can be found in the Github repository (<https://github.com/chaoruih/Clinical-Projects>)
